# Supplementary material for: Estimations of Mutation Rates Depend on Population Allele Frequency Distribution: The Case of Autosomal Microsatellites
Source: Genes (Basel). 2022 Jul 14;13(7):1248. doi: 10.3390/genes13071248 (PMC9323320; doi:10.3390/genes13071248)
Supplement: Supplementary file 1 [file genes-13-01248-s001.zip › File S1.pdf]

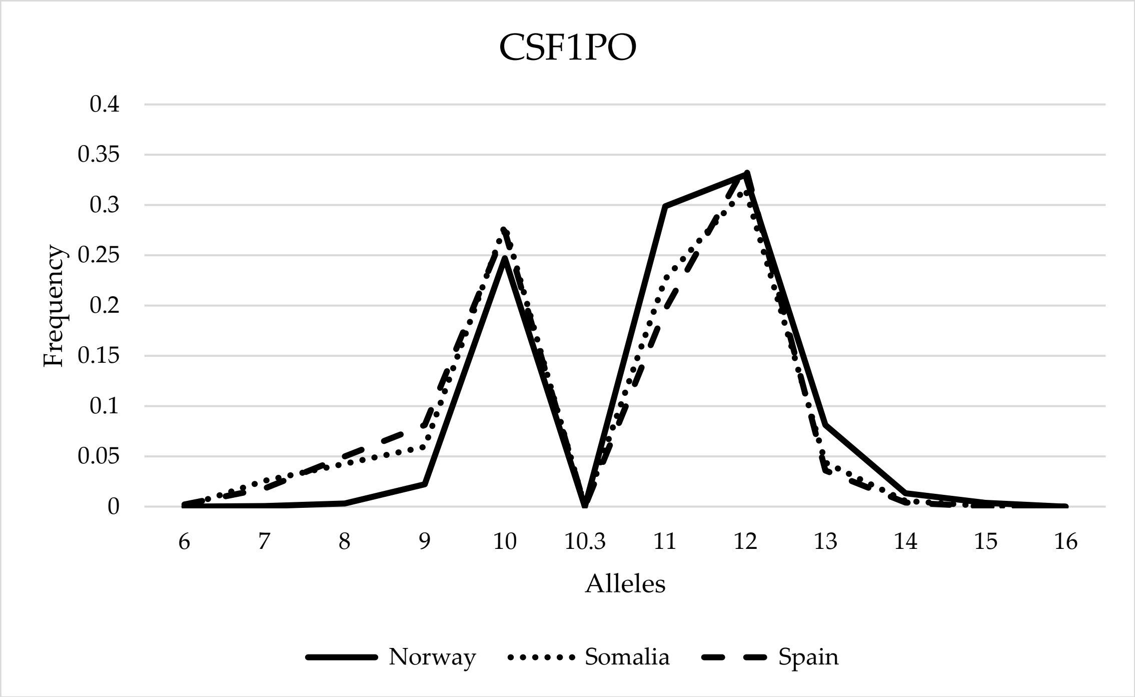

Figure 1. Allelic distribution of marker CSF1PO for the populations of Norway (N = 19156), Somalia (N = 1598) and Spain (N = 2500).

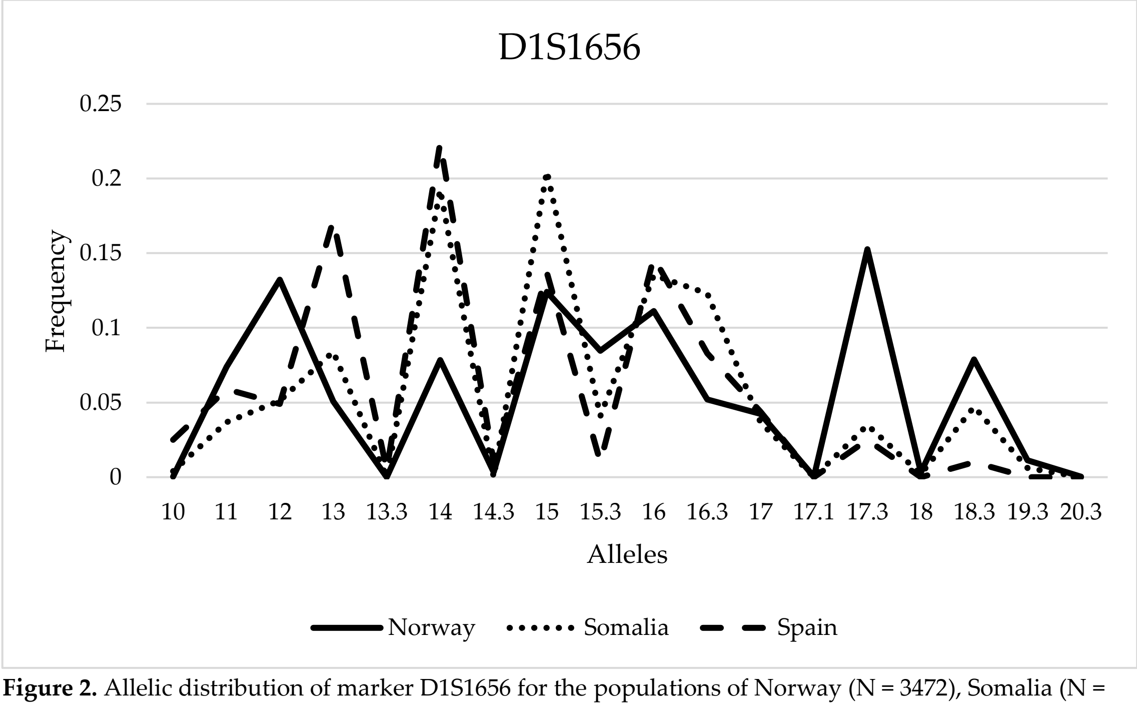

Figure 2. Allelic distribution of marker D1S1656 for the populations of Norway (N = 3472), Somalia (N = 488) and Spain (N = 2500).

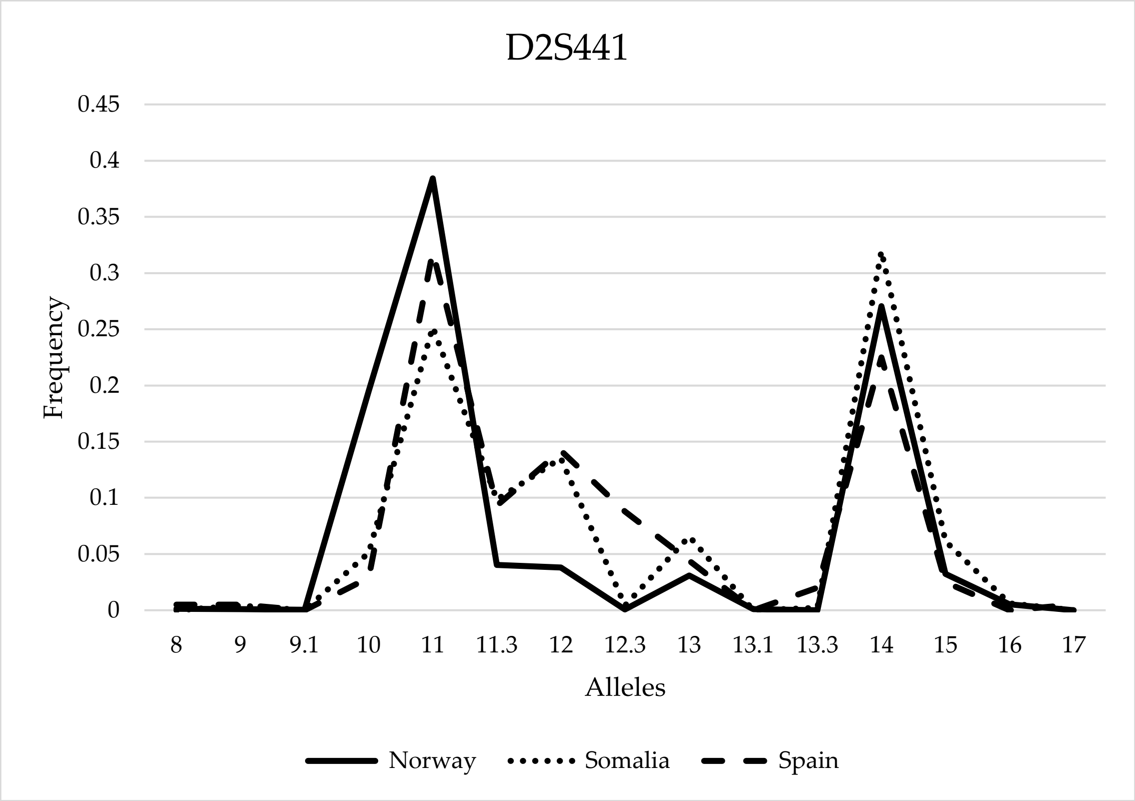

Figure 3. Allelic distribution of marker D2S441 for the populations of Norway (N = 3472), Somalia (N = 488) and Spain (N = 2500).

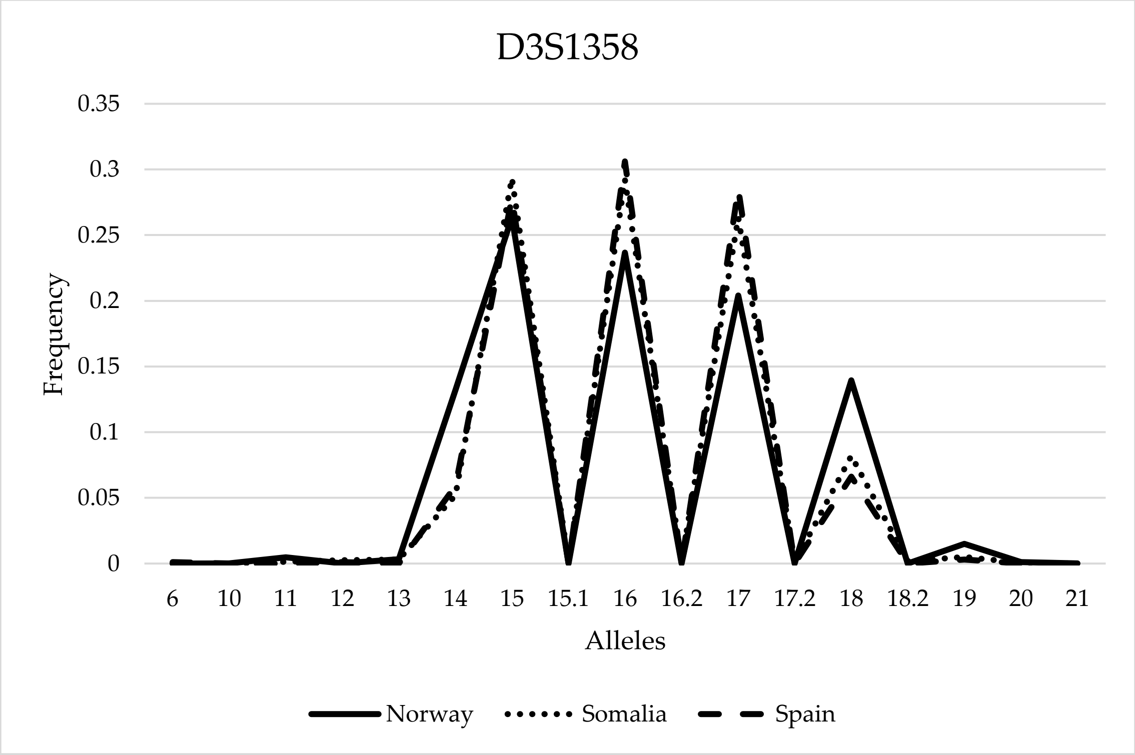

Figure 4. Allelic distribution of marker D3S1358 for the populations of Norway (N = 19172), Somalia (N = 1598) and Spain (N = 2500).

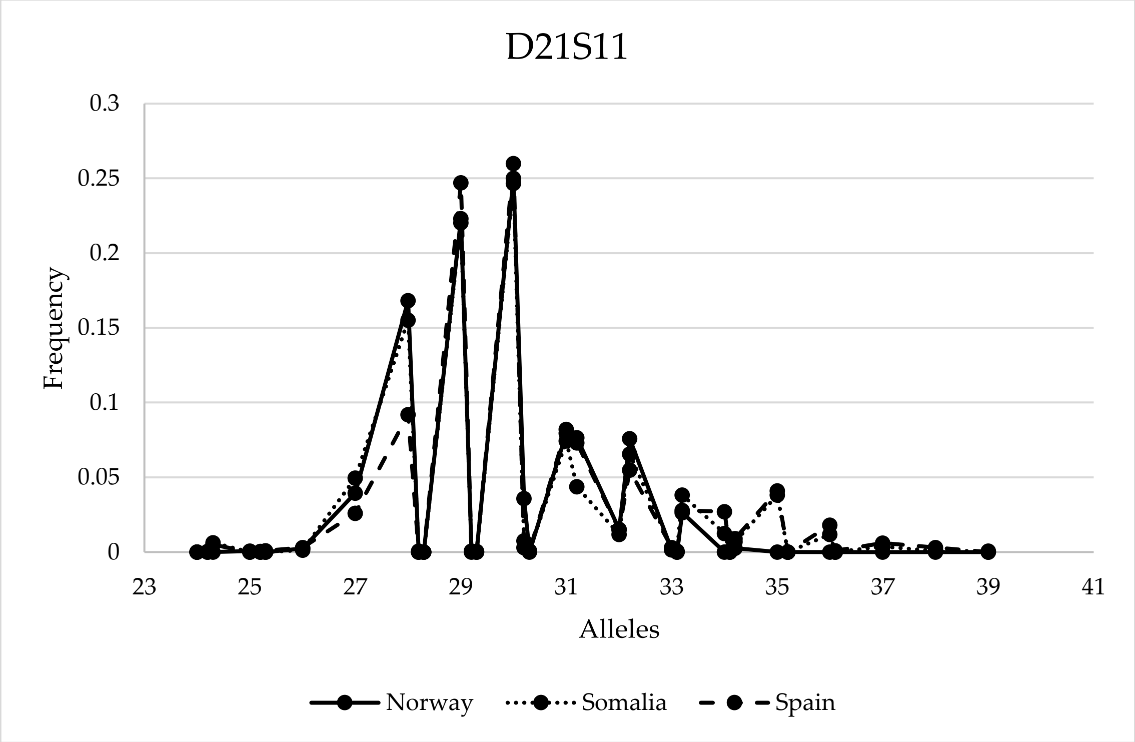

Figure 5. Allelic distribution of marker D21S11 for the populations of Norway (N = 19170), Somalia (N = 1598) and Spain (N = 2500).

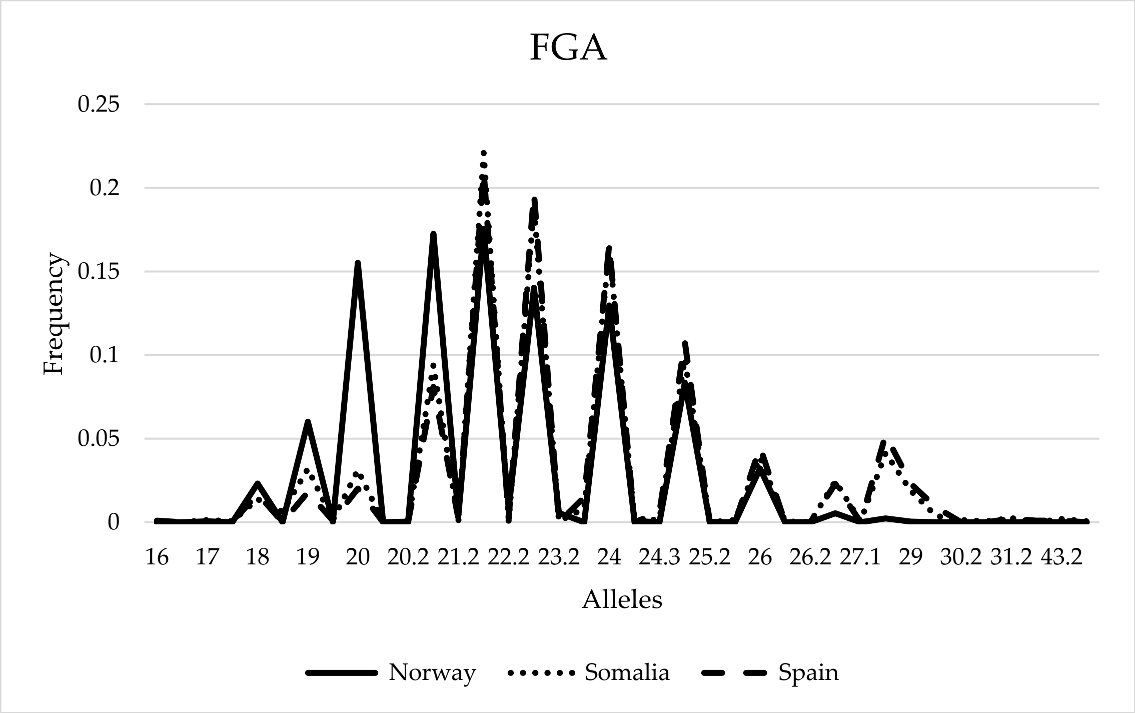

Figure 6. Allelic distribution of marker FGA for the populations of Norway (N = 19164), Somalia (N = 1598) and Spain (N = 2500).

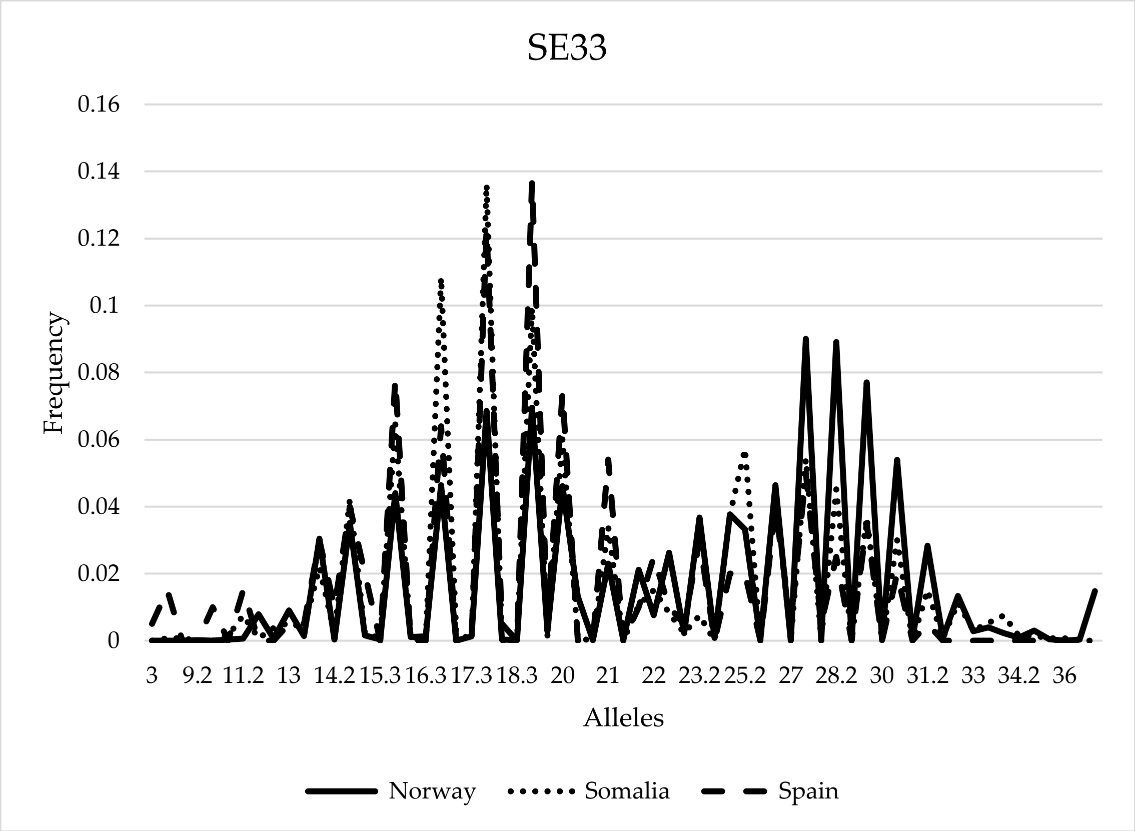

Figure 7. Allelic distribution of marker SE33 for the populations of Norway (N = 6318), Somalia (N = 1348) and Spain (N = 2500).

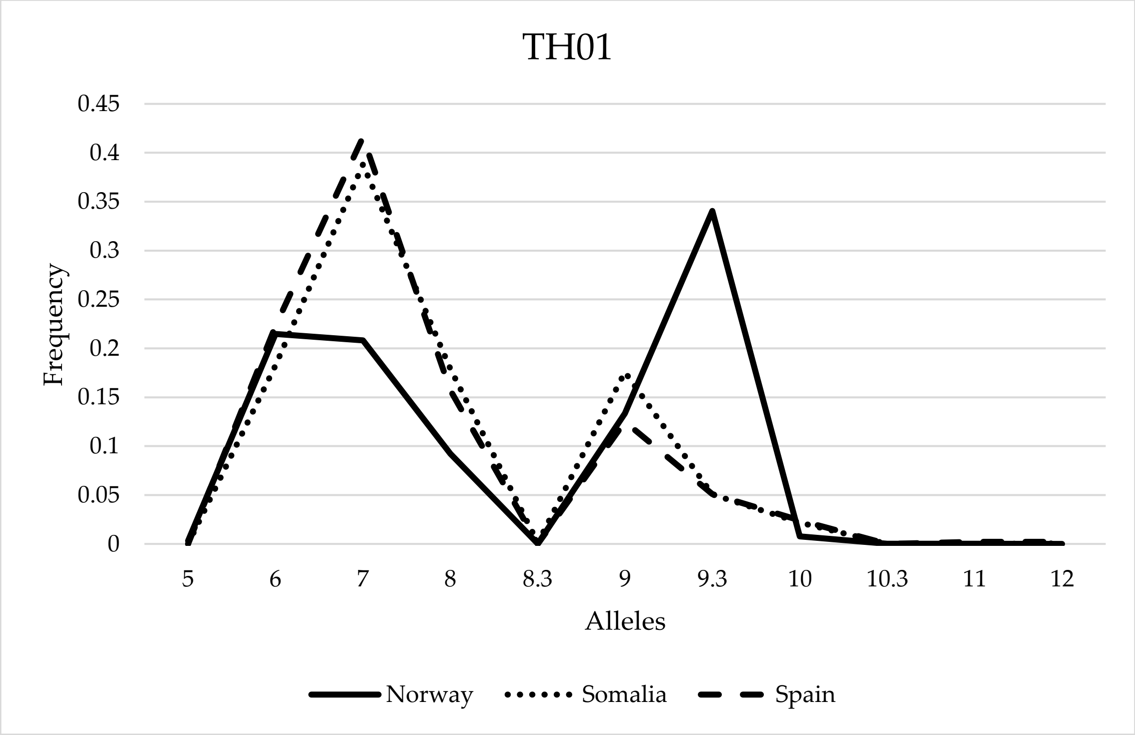

Figure 8. Allelic distribution of marker TH01 for the populations of Norway (N = 19172), Somalia (N = 1598) and Spain (N = 2500).

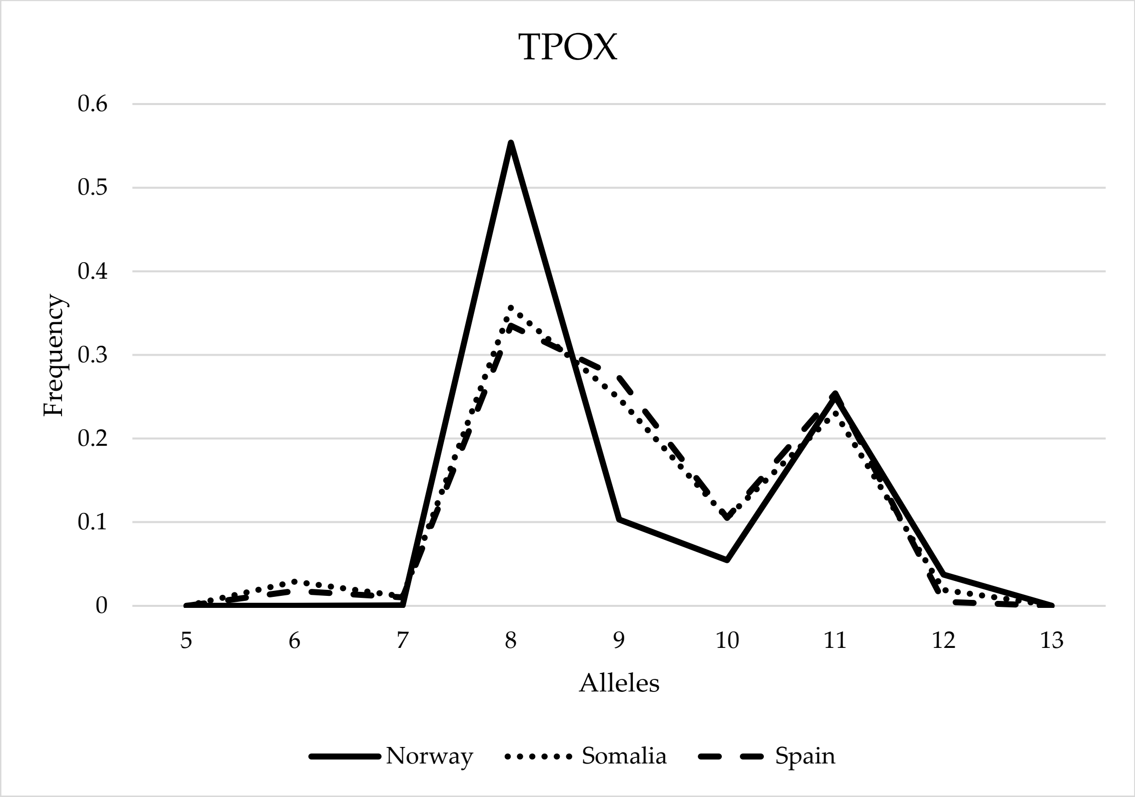

Figure 9. Allelic distribution of marker TPOX for the populations of Norway (N = 19162), Somalia (N = 1596) and Spain (N = 2500).

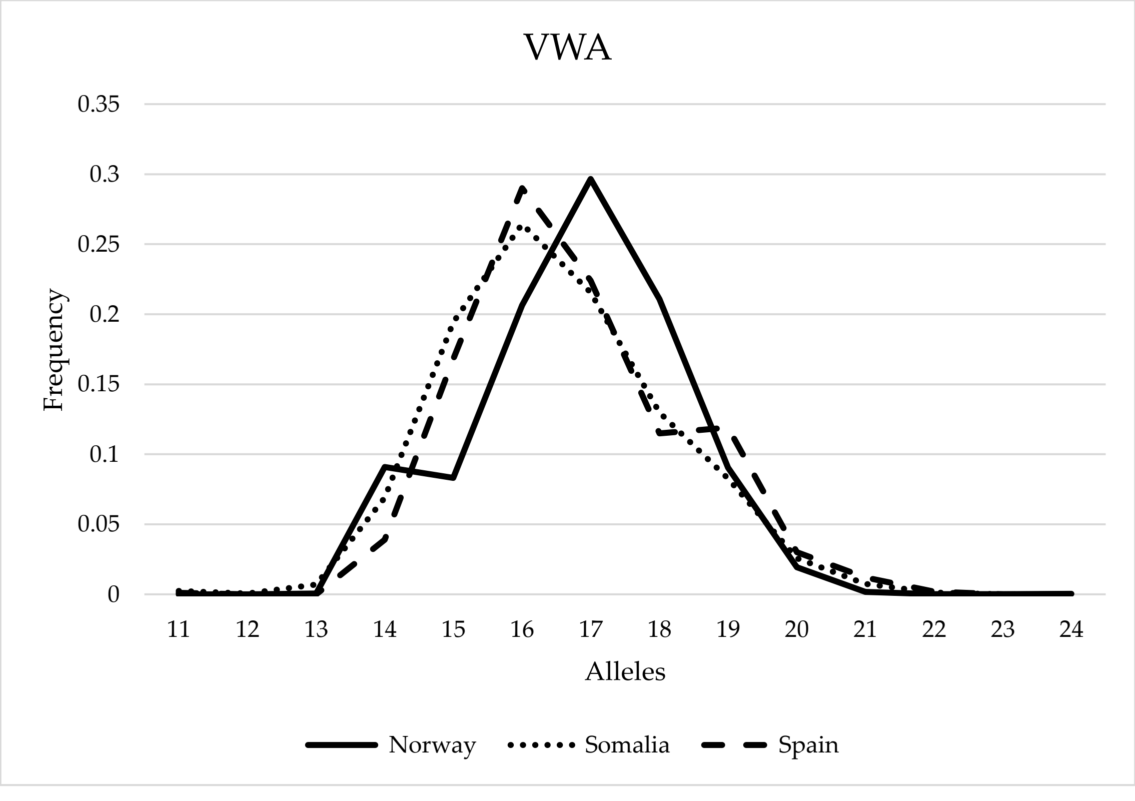

Figure 10. Allelic distribution of marker VWA for the populations of Norway (N = 19170), Somalia (N = 1597) and Spain (N = 2500).
