# Supplementary material for: Estimations of Mutation Rates Depend on Population Allele Frequency Distribution: The Case of Autosomal Microsatellites
Source: Genes (Basel). 2022 Jul 14;13(7):1248. doi: 10.3390/genes13071248 (PMC9323320; doi:10.3390/genes13071248)
Supplement: Supplementary file 1 [file genes-13-01248-s001.zip › Tables S1, S2 and S3.pdf]

Supplementary material

Table S1: Population size (N), expected heterozygosity (with Nei correction), polymorphic informative content (PIC), number of alleles and allelic range per marker and population.

Marker and population	N	Heterozygosity (Nei correction)	Polymorphic Informative Content (PIC)	Number of alleles	Allelic range
**CSF1PO (Norway)**	19156	0.7335	0.6860	8	9
**CSF1PO (Somalia)**	1598	0.7626	0.7250	7	8
**CSF1PO (Spain)**	2500	0.7609	0.7241	8	8
**D1S1656 (Norway)**	3472	0.8992	0.8905	9	10.3
**D1S1656 (Somalia)**	488	0.8699	0.8568	8	9.3
**D1S1656 (Spain)**	2500	0.8636	0.8493	8	8.3
**D21S11 (Norway)**	19170	0.8377	0.8186	10	11.2
**D21S11 (Somalia)**	1598	0.8489	0.8327	9	14.7
**D21S11 (Spain)**	2500	0.8432	0.8271	9	13.7
**D2S441 (Norway)**	3472	0.7361	0.6951	11	8
**D2S441 (Somalia)**	488	0.7955	0.7687	10	7
**D2S441 (Spain)**	2500	0.8072	0.7835	11	9
**D3S1358 (Norway)**	19172	0.7957	0.7644	12	11
**D3S1358 (Somalia)**	1598	0.7496	0.7065	12	9
**D3S1358 (Spain)**	2500	0.7397	0.6934	12	13
**FGA (Norway)**	19164	0.8666	0.8520	13	12.8
**FGA (Somalia)**	1598	0.8640	0.8501	14	28.2

**FGA (Spain)**	2500	0.8642	0.8501	12	27.2
**SE33 (Norway)**	6318	0.9479	0.9454	17	37.8
**SE33 (Somalia)**	1348	0.9336	0.9299	14	29.7
**SE33 (Spain)**	2500	0.9349	0.9313	14	28.2
**TH01 (Norway)**	19172	0.7683	0.7325	24	6
**TH01 (Somalia)**	1598	0.7501	0.7134	23	6
**TH01 (Spain)**	2500	0.7338	0.6962	22	6
**TPOX (Norway)**	19162	0.6157	0.5662	31	8
**TPOX (Somalia)**	1596	0.7458	0.7040	26	7
**TPOX (Spain)**	2500	0.7373	0.6907	25	6
**VWA (Norway)**	19170	0.8013	0.7736	46	11
**VWA (Somalia)**	1597	0.8167	0.7919	55	11
**VWA (Spain)**	2500	0.8075	0.7813	33	11

Table S2: Populations showing the highest and lowest standard deviations (σ between parentheses) considering the proportion of hidden mutations across markers, for each familial configuration type. A single-step mutation was simulated in one of the parental meiosis of 1,000,000 configurations of each type, considering the allele frequencies of 10 autosomal STRs in three populations (Norway, Somalia, and Spain) [30].

	**Duos**	**Trios**
**Average**	σ = 0.124	σ = 0.062
**Highest**	Norway (σ = 0.142)	Norway (σ = 0.067)
**Lowest**	Somalia (σ = 0.113)	Somalia (σ = 0.058)

Table S3: Average standard deviation and markers showing the highest and lowest values (in parentheses) of the proportion of hidden mutations across the different population databases: Norway, Somalia, and Spain, for each familial configuration type. A single-step mutation was simulated in one of the parental meiosis of 1,000,000 configurations of each type, considering the allele frequencies of 10 autosomal STRs in three populations (Norway, Somalia, and Spain) [30].

	**Duos**	**Trios**
**Average**	σ = 0.031	σ = 0.024
**Highest**	TPOX (σ = 0.061)	TH01 (σ = 0.073)
**Lowest**	FGA (σ = 0.004)	FGA (σ = 0.003)
